# Use Of A Unique Long Pre-curved Sheath To Facilitate Femoral Placement Of Coronary Sinus Catheters

**Published:** 2009-09-01

**Authors:** Adrian H Shandling, Daniel Rieders, Melanie Edwards

**Affiliations:** 1 University of California at Irvine; 2 Department of Electrophysiology, Memorial Medical Center, Long Beach, California

**Keywords:** coronary sinus cannulation, pre-curved sheath

## Abstract

**Methods:**

Catheterization of the coronary sinus (CS) from the femoral vein is widely used during electrophysiologic procedures.  Access to the CS may be difficult.  To address this problem we explored the utility of a long pre-formed (SAFL™) sheath in a cohort of consecutive patients requiring CS cannulation in the electrophysiology laboratory. This unique sheath has distal curvatures in 2 planes, potentially facilitating CS cannulation.

**Results:**

68 patients were studied with an average age of 63± 16 years.  In twelve patients (18%), standard femoral CS cannulation was ineffective.  In six of these patients, the SAFL™ sheath allowed for cannulation, and in six the subclavian approach was required.  There were no significant differences in age, left ventricular ejection fraction, or echocardiographically estimated pulmonary artery systolic pressure between the various subgroups. There was a trend towards a larger left atrial size in the atrial flutter group (46mm± 7.9) versus all others (40.6mm± 6.3, P=.076). Left atrial size was 37 mm in the femoral sheath-requiring group versus 44 mm in all others (P=NS).

**Conclusion:**

Utilization of a unique commercially available long preformed sheath helps to provide femoral CS catheter access in selected cases in the electrophysiology laboratory.

## Introduction

**Figure 1:**
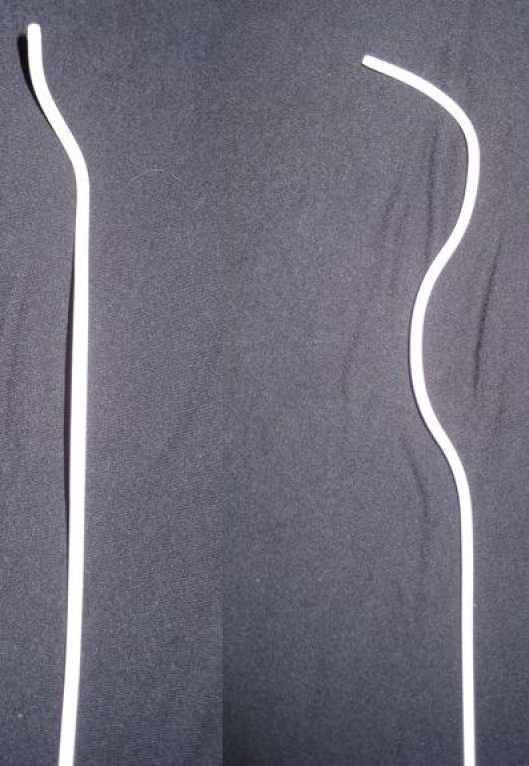
The terminal portion of the SAFL™ sheath is shown in two right–angled views to demonstrate the unusual three-dimensional shape of the terminal portion of the catheter.  The double offset pictured helps to direct the catheter tip posteriorly towards the CS os. The lower curve provides extra stability in the IVC.

Catheterization of the coronary sinus (CS) from the femoral vein is widely used during electrophysiologic evaluation. The technique has been previously reported [1]. The alternate approach using subclavian or jugular access has potential for complications, such as pneumothorax and inadvertent arterial puncture. Problems with the femoral approach relate more to the variability of the CS origin and its course, rather than a risk of appreciable complications [2] [3]. There are also differences in inferior vena cava (IVC) size and right atrial size, contributing to a lack of catheter support and further cannulation difficulties. Catheter placement can be challenging. To help mitigate the problem associated with femoral CS cannulation, we initiated the use of a commercially available preformed long ablation sheath (St. Jude Medical Fast-Cath™ with a SAFL™ curve). This sheath was specifically designed for typical right atrial flutter ablations, but it uniquely has curvatures in two, rather than the usual single plane (Figure 1). It is utilized by the authors if the CS is not rapidly and effectively cannulated from the femoral approach. We analyzed our success for femoral CS cannulation alone or utilizing this sheath technique in a consecutive series of patients. Echocardiographic variables were also analyzed to assess if there was any factor that could predict failure of standard femoral CS cannulation.

## Methods

From January 2004 to September 2005 we analyzed the frequency of use of the SAFL™ sheath for facilitation of femoral CS cannulation. Included were consecutive cases by one operator requiring coronary sinus access in one electrophysiology laboratory. Five minutes of biplane fluoroscopy time was spent attempting to cannulate the CS from the femoral route. Either a 5 French octapolar or 6 French decapolar deflectable tip catheter was used in the sheath. If unsuccessful, or the catheter was unstable, or could not be advanced sufficiently far down the CS, a SAFL™ sheath was passed up via the femoral vein. Another five minutes of biplane fluoroscopy time was spent in the attempt at CS cannulation through the sheath. If this failed, subclavian access was obtained for the placement of the CS catheter. Parameters were recorded from echocardiographic studies performed within six months of the ablation. Statistical analysis was performed utilizing chi square analysis and Student's t test with a P value of < 0.05 significant. The data was retrospectively collected so there was no institutional review board involvement as per our protocol.

## Results

A total of 68 Cases were analyzed.  The average age of the study cohort was 63± 16 years of age. There were no procedural complications related to placement of the CS catheters, subclavian catheters, or sheaths. Conventional femoral access was obtained in 56 patients (82%).  In six cases (9%), a SAFL™ sheath was necessary, and subclavian approach was required in six cases (9%).  There were 37 typical right atrial flutter cases (average age of 70± 12 years).  The other 31 cases (average age 57± 18 years) included accessory pathways, atrial tachycardia, AV nodal re-entrant tachycardia, and atrial fibrillation (1 case). The average left ventricular (LV) ejection fraction was 59%± 13.  No significant difference was seen when the LV ejection fraction, LV chamber size and LV thickness were compared between the sheathless group of 56 and the 12 cases requiring either the sheath or subclavian approach. Left atrial size was 37mm in the sheath-requiring group versus 44mm in all others (P=NS).  Left atrial size was not significantly larger in the atrial flutter group (46mm± 7.9) versus all others (40.6mm± 6.3, P=.076).  An estimated pulmonary artery systolic pressure was available in 46 patients (33mm/Hg± 11). Pressures were similar in all sub-groups.

## Discussion

Catheterization of the coronary sinus is increasingly important in electrophysiology today. It is used diagnostically and therapeutically for intracardiac ablation cases and is used therapeutically for placement of "left ventricular" pacemaker leads. The initial description of the femoral technique was reported in 1995 [1]. The catheterization success rate in this report was 97%, compared with 73% in our study. However, this high success was achieved before 1995, when the diagnosis in the majority of cases was an accessory pathway or atrioventricular nodal re-entrant tachycardia. Patients were younger and hearts likely morphologically normal. There are a number of previous reports concerning long sheaths [4-6]. These are generally sheaths that are not commercially available and do not have the three-dimensional curve of the catheter in the present study. Success rates for placement in these studies were high, but again, this was predominantly work done in the era of cases with structurally more normal hearts. Current ablations for atrial flutter and fibrillation can involve patients with appreciably abnormal heart sizes and positions, resulting in an unusually positioned or abnormally directed CS os [2,7]. The Thebesian valve can cover the os and is oriented anterosuperiorly, affecting a femorally placed catheter to a greater degree [3]. Fenestrations may overly the CS os. The valve of Vieussen can also be prominent proximally in the CS and impede passage of a catheter [3].

We now use a St. Jude Medical Fast-Cath™ with a SAFL™ curve routinely in difficult cannulation cases (usually recommended for isthmus-dependent atrial flutter ablations to provide ablation catheter stability). The sheath is placed along the posterior intra-atrial septum and provides support and helps direct the diagnostic catheter posteriorly toward the CS os. An octapolar or decapolar deflectable tip catheter is used for CS cannulation and recording. The extra support and shape of the sheath allows for entry and deeper catheter penetration into the CS. The usual electrophysiology lab approach after a failed femoral attempt is to access the subclavian or jugular vein. The additional time required to "prep" the subclavian area for catheter placement from this site may be saved. The sheath has not been an obstacle to subsequent right-sided ablation catheter positioning.

The SAFL™ sheath was used in a relatively small number of cases in this series and this does limit statistical analysis. It does not however, detract from the utility of this technique in cases where CS cannulation is difficult. Futhermore, the technique may have a wider application. CS catheter stability has assumed increased importance since the advent of electro-mechanical mapping, which requires immutability of a reference point during mapping. The sheath may provide more stability than a catheter alone, preventing catheter-slide and thereby improving three-dimensional map quality.
